# Colostrum of Healthy Slovenian Mothers: Microbiota Composition and Bacteriocin Gene Prevalence

**DOI:** 10.1371/journal.pone.0123324

**Published:** 2015-04-28

**Authors:** Tanja Obermajer, Luka Lipoglavšek, Gorazd Tompa, Primož Treven, Petra Mohar Lorbeg, Bojana Bogovič Matijašić, Irena Rogelj

**Affiliations:** 1 Institute of Dairy Science and Probiotics, Department of Animal Science, Biotechnical Faculty, University of Ljubljana, Domžale, Slovenia; 2 Department of Animal Science, Biotechnical Faculty, University of Ljubljana, Domžale, Slovenia; Center for Molecular Biotechnology, ITALY

## Abstract

Microbial communities inhabiting the breast milk microenvironment are essential in supporting mammary gland health in lactating women and in providing gut-colonizing bacterial 'inoculum' for their infants’ gastro-intestinal development. Bacterial DNA was extracted from colostrum samples of 45 healthy Slovenian mothers. Characteristics of the communities in the samples were assessed by polymerase chain reaction (PCR) coupled with denaturing gradient gel electrophoresis (DGGE) and by quantitative real-time PCR (qPCR). PCR screening for the prevalence of bacteriocin genes was performed on DNA of culturable and total colostrum bacteria. DGGE profiling revealed the presence of *Staphylococcus* and *Gemella* in most of the samples and exposed 4 clusters based on the abundance of 3 bands: *Staphylococcus epidermidis/Gemella*, *Streptococcus oralis/pneumonia* and *Streptococcus salivarius*. *Bacilli* represented the largest proportion of the communities. High prevalence in samples at relatively low quantities was confirmed by qPCR for enterobacteria (100%), *Clostridia* (95.6%), *Bacteroides-Prevotella* group (62.2%) and bifidobacteria (53.3%). Bacterial quantities (genome equivalents ml^-1^) varied greatly among the samples; *Staphylococcus epidermidis* and staphylococci varied in the range of 4 logs, streptococci and all bacteria varied in the range of 2 logs, and other researched groups varied in the range of 1 log. The quantity of most bacterial groups was correlated with the amount of all bacteria. The majority of the genus *Staphylococcus* was represented by the species *Staphylococcus epidermidis* (on average 61%), and their abundances were linearly correlated. Determinants of salivaricin A, salivaricin B, streptin and cytolysin were found in single samples. This work provides knowledge on the colostrum microbial community composition of healthy lactating Slovenian mothers and reports bacteriocin gene prevalence.

## Introduction

Breast milk secreted within the first 3 days after childbirth is one of the first essential environmental factors that shape the life of a neonate. While acting as a complex and multifunctional nutrition source, it provides immunological benefits and a gut-colonizing bacterial 'inoculum' for the infant's gastro-intestinal (GIT) development [1].

The largest compositional variation in human milk is due to the stage of lactation [2]. The colostrum's compositional complexity and compartmentalization (aqueous phase, colloidal dispersion, emulsion and host cells) form an environment consistent with the development of a delicate microbial community. At the end of pregnancy, the increased blood and lymph supply to the mammary gland and released oxytocin enable endogenous bacteria to inhabit breast milk, most likely by forming biofilms on the mammary areola or duct system [1, 3, 4], creating a specific and transitory mammary microbiota. Biofilms result from a fine balance between competition and cooperation, which can be upset by a variety of influences in the surrounding environment and quorum sensing-dependent gene regulation [5]. The milk microbiome is subjected to constant shaping by pre- and postnatal factors, such as physiological changes triggering maternal gut permeability and changes induced by interaction with the mother's skin microbiota and with the infant's oral microbiota during suckling [6]. A recent study performed by Jost et al. [7] on seven mother-neonate pairs confirmed the potential for breast milk to mediate the colonization of the neonate gut with maternal gut bacteria. Gut-associated obligate anaerobes (*Clostridia* members, *Bifidobacterium*, *Bacteroides*, and *Parabacteroides*) were shared between the three ecosystems (maternal feces, breast milk and neonatal feces). 'Active migration' of bacteria from the maternal gut has been proposed to be involved in establishing the mammary gland community, but this hypothesis is as yet unconfirmed. Antimicrobial peptides, or bacteriocins, offer their producers a competitive advantage in niche settlement [8]. Lakshminarayanan et al. [9] characterized culturable bacteriocin producers, lactic acid bacteria (LAB) and other bacteria, in the intestinal microbiota. Because bacteriocin producers may modulate the intestinal ecology, this advantage may also apply to the ecosystem of the mammary gland in lactating women and influence the infant GIT formation. Hunt et al. [10] suggested that complex, diverse and individually unique breast milk microbiota may also play a beneficial role in the context of mammary health in lactating women by preventing gland infections and inflammation. Certain commensal milk bacteria or probiotic bacteria might repress the growth of host pathogens by producing bacteriocins and other bioactive substances or by competing for nutrients and resources. Ward et al. [11] reported the diverse functional capabilities of the microbiome of pooled milk from ten donors, and 4.5% of all the microbiome open reading frames (ORFs) were associated with virulence, disease and defense; of those ORFs, 2.7% were assigned to bacteriocins, impacting the growth of other microbes. Thus, bacteria residing within human milk may be part of the endogenous immune system of milk.

The colostrum microbiota has been investigated previously [12–15]. Cabrera-Rubio et al. [12] reported bacterial diversity to be higher in colostrum than in transitional milk or in mature milk. These investigators revealed that the common genera in colostrum samples of 18 mothers were lactic acid bacteria (*Weissella*, *Leuconostoc*, *Streptococcus*, *Lactococcus*) and *Staphylococcus*. The authors suggested that the mothers' geographic origin and environmental factors could have influenced the results obtained. Collado et al. [16] assessed the bacterial diversity of breast milk by qPCR and affirmed the utility of this culture-independent method for microbiological analyses of human milk. The interactions of species in defined multispecies biofilm formation have also been evaluated by qPCR, and a standard protocol has been suggested for natural settings where species diversity is more restricted or when limited key species are in the research focus [17]. The PCR-DGGE approach has been successfully applied to evaluate the total microbial diversity of human milk and to analyze breast milk lactobacilli [18, 19]. In this study, the colostrum microbiome was investigated in 45 healthy Slovenian lactating mothers. The composition of the microbiota in the colostrum of different subjects was assessed by PCR-DGGE fingerprinting of the V3 16S rRNA region. Quantitative evaluations of staphylococci, streptococci and bacterial groups commonly found in feces (*Bacteroides-Prevotella*, clostridia, enterobacteria, bifidobacteria, lactobacilli, and enterococci) were conducted by qPCR. Quantitative associations of the detected bacterial groups were investigated. PCR screening for the prevalence of bacteriocin genes typical of LAB and some other bacteria was performed on the total microbial DNA of colostrum and on DNA of colostrum bacterial consortia grown on MRS and M17 agar media.

## Materials and Methods

### Study design and sampling

The research was conducted as part of the observational clinical study registered at ClinicalTrials.gov (http://clinicaltrials.gov/ct2/show/record/NCT01548313). The study was approved by The National Medical Ethics Committee of the Republic of Slovenia (32/07/10; 38/02/12), and all of the participating mothers provided written informed consent. All data on the subjects were coded, and the information was kept confidential. Non-invasive, participant-friendly procedures were used for biological sample collection. All study volunteers were under medical observation and were offered support during the whole course of the study while having access to their personal data.

Healthy Slovenian pregnant women in the first two trimesters of pregnancy (maximum 24 weeks gestation) from the Ljubljana, Maribor and Izola regions who planned to breast-feed their babies for at least 6 weeks after birth were included in this research. The exclusion criteria were pregnancy in the last trimester, diagnosis of autoimmune chronic disease, acute or chronic infections or an increased risk of early birth.

On the 2^nd^ or 3^rd^ day after delivery, participating mothers at maternity hospitals (Ljubljana, Maribor and Izola) collected colostrum samples. Approximately 1–2 ml of colostrum was aseptically collected by manual expression from each breast under the supervision of the medical staff to reduce the risk of sample contamination with skin bacteria. Briefly, hands and breasts were first cleaned well with antiseptic soap and a sterile physiological solution. Afterwards, the breasts were carefully dried with sterile paper towels, especially in the area of the areolas and nipples. Milk secretion was facilitated by gentle circular massage, and the first drops of colostrum were discarded. Finally, a few milliliters of colostrum was collected from the left and right breasts separately in sterile containers. The samples were immediately frozen and kept at -20°C. Within a month, the samples were transferred to the laboratory and were stored at -80°C for up to a few months until the analysis was performed.

### Colostrum—sample preparation for PCR-DGGE, qPCR and PCR bacteriocin detection analyses

Colostrum samples obtained from the left and right breasts of a single mother were mixed together in an equal ratio. Then, approximately 2 ml of this mixed sample was pelleted by centrifugation (3600 g/10 min/4°C). The supernatant with the fat layer was removed, and the pellet was kept frozen (-20°C) until DNA extraction. Lysis of the pelleted bacterial cells was conducted by a 2 hour incubation with lysozyme (5 mg/ml) and mutanolysin (5 U/ml) before the samples were sonicated (Soniprep 150 plus, MSE Limited, London, UK) and transferred to a Maxwell 16 Tissue DNA Purification Kit cartridge (Promega, Madison, WI, USA). DNA was extracted according to the automated Maxwell 16 System (Promega) protocol. Finally, approximately 200 μl of eluate was obtained. The eluates were further submitted to NanoVue (GE Healthcare Life Sciences, Uppsala, Sweden) spectrophotometrical evaluation and PCR with a broad-range bacterial primer set to confirm the presence of bacterial DNA.

### Colostrum viable consortia preparation for PCR bacteriocin detection

The colostrum samples were homogenized and diluted in quarter-strength Ringer’s solution. Non-diluted and 1:10 diluted samples were spread on M17 and MRS (Merck, Darmstadt, Germany) agar plates and incubated at 37°C (48–72 hours) aerobically (M17) or anaerobically (MRS) (GENbox anaer, Bio-Mérieux, Marcy l’Etoile, France). Countable plates with no more than 300 colonies were rinsed from the surface of the MRS and M17 agar plates with 2 ml of quarter-strength Ringer’s solution and were centrifuged (3600 g for 10 min at 10°C) [20]. The pellet was resuspended in 600 μl of 50 mM EDTA containing 0.01 mg μl^-1^ lysozyme and 0.025 U μl^-1^ mutanolysin and was incubated at 37°C for 1 hour. DNA was extracted from the pelleted bacteria using the Wizard Genomic DNA Purification kit (Promega, Madison, WI, USA) following the manufacturer’s instructions. The efficiency of the DNA extraction was verified spectrophotometrically (NanoVue).

### PCR DGGE fingerprinting

PCR-DGGE fingerprinting was performed to investigate the differences in colostrum microbiota composition. The HDA1-GC and HDA2 primer sets [21] were used to amplify the V3 region of bacterial 16S rRNA.

PCR reactions were accomplished in a 25 μl mixture composed of 0.625 U of *Taq* DNA polymerase (GoTaq Flexi, Promega, Madison, WI, USA), 1x Colorless GoTaq Flexi buffer, each deoxynucleoside triphosphate at a concentration of 200 μM, 2.5 mM MgCl_2_, primers at a concentration of 0.5 μM each and 1 μl of DNA template. The reactions proceeded in a Gene Amp 2700 (Applied Biosystems, Carlsbad, CA, USA) using the following program: initial denaturation (2 min at 95°C), 35 cycles of amplification (95°C for 30 s, 58°C for 30 s, and 72°C for 40 s) and a final elongation (72°C for 5 min).

Analysis of the PCR amplicons was conducted with the D GENE denaturing gel electrophoresis system (Biorad, Hercules, CA, USA). The denaturing gradient gel consisted of 8% polyacrylamide (acrylamide:bis-acrylamide = 37:1, Sigma-Aldrich, Saint Louis, MO, USA) and 30 to 65% denaturants (urea and formamide). The electrophoresis was run at 60°C and 75 V for 16 h. The gels were stained with SYBR Safe (Invitrogen, Carlsbad, CA, USA) and visualized with UV transillumination and a short-wave band pass filter using ChemiGenius2 (Syngene, Cambridge, UK).

The identity of selected bands was determined by excising the bands from DGGE gels, followed by PCR amplification and the subsequent sequencing of the PCR products (Microsynth, Balgach, Switzerland). Sequence identity was established using the RDP Classifier tool [22] and phylogenetic trees including similar sequences from the SILVA 115 database [23].

### QPCR analysis

#### Preparation of standards

Standards for the qPCR analyses were prepared from bacterial genomic DNA isolated from overnight pure bacterial cultures of *Streptococcus salivarius* K12, *Staphylococcus epidermidis* IM 385, *Enterococcus faecalis* DSM 20478, *Lactobacillus gasseri* K7 IM 105, *Bifidobacterium animalis* subsp. *lactis* BB-12, *Bacteroides thetaiotaomicron* DSM 2079, *Clostridium clostridioforme* DSM 933, *Clostridium leptum* DSM 753 and *Escherichia coli* K12. The bacterial cultivation, cell number determination and operon copy number estimations were performed as previously described by Matijašić et al. [24] for 16S rRNA gene targets and for additional targets, as listed in Table 1. DNA was extracted from bacterial cells as described above for colostrum.

**Table 1:** Cultivation media, conditions and copy number estimates of traced bacterial operons in the standard curve calibration.

Bacterial strain ^a^	Medium ^b^	Cultivation condition	Copy number estimates (traced operons) ^c^
*Streptococcus salivarius* K12 (BLIS Technologies Ltd, Dunedin, NZ)	BHI		1 (TUF gene)
*Staphylococcus epidermidis* IM 385	BHI	aerobic/37°C/18 h	1 (DNAJ/TUF gene)
*Enterococcus faecalis* DSM 20478	M17		4 (16S rRNA gene)
*Lactobacillus gasseri* K7 IM 105	MRS		6 (16S rRNA gene)

^a^IM *Microbial Collection of Institute of Dairy Science and Probiotics*, Biotechnical Faculty, University of Ljubljana, Slovenia

^a^DSM *Deutsche Sammlung von Mikroorganismen und Zellkulturen*, Braunschweig, Germany

^b^BHI Brain Heart Infusion Broth (Merck, Darmstadt, Germany)

^b^M17 Broth acc. to TERZAGHI (Merck, Darmstadt, Germany)

^b^MRS de Man, Rogosa and Sharpe Medium (Merck, Darmstadt, Germany)

^c^ 16S rRNA operon copy numbers of target species were derived from the rrnDB database

#### Quantitation of colostrum DNA

Specific microbial quantitation analysis in our study was based on the conserved marker gene 16S rRNA for all bacterial groups, except for *Staphylococcus*, *Streptococcus* and *Staphylococcus epidermidis*, where alternative marker genes were used instead (Table 1).

Real-time PCR quantitation of the target gene copy number in colostrum DNA was conducted with the Stratagene Mx3000P instrument (Stratagene, La Jolla, CA, USA). The reactions were carried out in a total volume of 20 μl comprising KAPA SYBR Fast Master Mix (2x) Universal (KapaBiosystems, Boston, USA), 0.2 μM each of the two oligonucleotide primers (specified in Table 2), 5 μl of colostrum DNA (10-fold diluted) and 5 μl of sterile Milli-Q water for the non-template controls (NTCs). The amplification efficiencies of randomly selected samples and standards were tested in different target reactions; as recommended, the efficiencies were within 5% of each other. In each experiment, at least 6 dilutions (1:2) of standard DNA were included in different assays covering a linear dynamic range from approximately 3 to 6 logs. Standard curves representing the correlation between Ct values and the numbers of bacterial copies were generated by the Stratagene software Mx3000P, and sample concentrations (number of copies/ml of colostrum) were obtained from the cycler’s system program interpolating the sample Ct value into the standard curve. Reaction efficiency was calculated from the slope of the standard curve (Table 2). Samples and NTCs were run in duplicate, and two runs were conducted for each primer set. For most of the samples, the ΔCt between duplicates was in the range of ≤ 0.5 cycles. The theoretical limit of detection (LOD), considering at least 3 copies per PCR reaction, was calculated from the equation of the standard curve using the last cycle number as the cut off value. For all bacteria primer pairs, the NTCs crossed the threshold before the last cycle (35); however, the sample quantification frame was still reliable because no investigated samples crossed the threshold less than 3.3 cycles before the first NTC. Finally, a melting curve analysis was performed to verify the similarity of the curve profiles of standards and samples. For the purpose of statistical analysis, a value corresponding to half of the LOD was assigned to results below the LOD of qPCR assays. Additionally, the detected copy numbers were converted to genome equivalents (GE) (as presented in Table 2) based on the available rrnDB data on the average 16S rRNA gene copy number per target genome for the closest taxonomic group available and on *tuf* gene copy number references [25, 26].

**Table 2:** Organisms and their average target copies per genome, investigated by quantitative PCR analysis, with respective oligonucleotide primer pairs, reaction conditions and parameters.

Target organism	Target region	Average values for copies 16S rRNA/ target genome (rrnDB)	Primer set	Primer Sequence (5' to 3')	Product size (bp)	Annealing temp. (°C)/ time (sec)	Reference	Quantitative PCR Parameters of 2 Runs	
								R^2^; Efficiency	Limit of Detection (copies per reaction mix)
All bacteria	16S rDNA	4.2	Eub338F	ACTCCTACGGGAGGCAGCAG	200	53/30	[44]	0.997; 96.8%	10.4
		(Domain: Bacteria)	Eub518R	ATTACCGCGGCTGCTGG				0.998; 96.1%	16.1
*Bacteroides-Prevotella* group	16S rDNA	6	Bac303F	GAAGGTCCCCCACATTG	418	62/30*	[45–46]	0.998; 101.1%	3.5
		(Family: Bacteroidaceae)	Bac 708R	CAATCGGAGTTCTTCGTG				0.998; 101.3%	3.1
*Bifidobacterium*	16S rDNA	3.56	Bif-F	TCGCGTC(C/T)GGTGTGAAAG	243	59/15*	[47]	0.997; 89.6%	4.5
		(Genus: Bifidobacterium)	Bif-R	CCACATCCAGC(A/G)TCCAC				0.994; 89.9%	4.9
*Clostridium* cluster IV (*leptum* group)	16S rDNA	7.2	S-*-Clos-0561-a-S-17	TTACTGGGTGTAAAGGG	580	60/60	[48]	0.996; 99.9%	25.9
		(Order: Clostridiales)	S-*-Clept-1129-a-A-17	TAGAGTGCTCTTGCGTA				0.994; 99.9%	5.9
*Clostridium* cluster XIV (*coccoides* group)	16S rDNA	7.2	g-Ccoc-F	AAATGACGGTACCTGACTAA	ca. 440	50/20	[49]	1.000; 90.6%	5.9
		(Order: Clostridiales)	g-Ccoc-R	CTTTGAGTTTCATTCTTGCGAA				0.992; 90.9%	16.5
*Enterobacteriaceae* group	16S rDNA	6.16	Eco 1457f	CATTGACGTTACCCGCAGAAGAAGC	195	63/30	[45]	0.995; 92.2%	3.5
		(Family: Enterobacteriaceae)	Eco 1652r	CTCTACGAGACTCAAGCTTGC				0.999; 92.1%	3.4
*Staphylococcus*	*tuf* gene	NA**	TStaG422	GGCCGTGTTGAACGTGGTCAAATCA	370	58/30	[50]	0.997; 91.3%	24.9
		(1 copy per genome)	TStag765	TIACCATTTCAGTACCTTCTGGTAA				0.998; 91.1%	10.5
*Staphylococcus epidermidis*	*dnaJ* gene	NA	J-StGen	TGGCCAAAAGAGACTATTATGA	249	60/30	[34]	0.998; 97.0%	3.7
		(1 copy per genome)	J-StEpi	CCACCAAAGCCTTGACTT				0.998; 97.0%	5.8
*Streptococcus*	*tuf* gene	NA**	Tuf-Strep-1	GAAGAATTGCTTGAATTGGTTGAA	560	62/30	[16]	0.999; 90.1%	5.5
		(1 copy per genome)	Tuf-Strep-R	GGACGGTAGTTGTTGAAGAATGG				1.000; 89.9%	5.1
*Lactobacillus* (including *Leuconostoc*, *Pediococcus*, *Weissella)*	16S rDNA	5.24	LactoR'F	CACAATGGACG(A/C)AAGTCTGATG	358	65/15	[51]	0.996; 89.9%	23.6
		(Order: Lactobacillales)	LBFR	CGCCACTGGTGTTCTTCCAT				0.998; 89.6%	7.1
*Enterococcus*	16S rDNA	4.67	Enc-F	CCCTTATTGTTAGTTGCCATCATT	144	61/20	[47]	0.997; 92.9%	24.0
		(Genus: Enterococcus)	Enc-R	ACTCGTTGTACTTCCCATTGT				0.997; 92.9%	31.1

*modified from the reference

** *tuf* gene gene copy number reference [25–26]

### PCR screening for bacteriocin genes

PCR screening for 25 bacteriocin genes (S1 Table) was performed on bacterial DNA from colostrum and on DNA obtained from culturable bacterial consortia grown on MRS and M17 agar plates. The PCR amplifications were carried out in a SureCycler 8800 (Agilent Technologies, Santa Clara, CA, USA). The reactions were accomplished in a total volume of 20 μl, and the mixture comprised 1 μl of DNA (colostrum or consortia) or 1 μl of sterile Milli-Q water for the negative reaction control, GoTaq Buffer including 1.5 μM MgCl_2_ and 0.5 U GoTaq Flexi DNA Polymerase (Promega, Madison, WI, USA), 100 μM dNTPs (Thermo Fisher Scientific, Waltham, MA, USA) and the appropriate 1 μM primer pair (Invitrogen, Carlsbad, CA, USA) (S1 Table). For plantaricin A [27], 25 mM MgCl_2_ was added to achieve the final reaction concentration of 2.5 mM. Amplified fragments were resolved by electrophoresis on 1.8% (w/v) agarose gels and stained with the SYBR Safe DNA gel stain (Invitrogen). The specificity and identity of the PCR amplicons were determined by sequence analysis (Microsynth AG, Balgach, Switzerland) after purification with the Wizard SV Gel and PCR Clean-Up System (Promega). The sequence homology was analyzed by comparison with sequences in the NCBI database using BLAST.

Sequenced amplicons of bacteriocin genes from various dairy origins and DNA of the following reference strains were applied as positive controls in the PCR reactions: *Enterococcus faecium* LMG 11423 (strain that produces enterocins A, B and P; purchased from BCCM/LMG, Gent, Belgium), *Lactobacillus helveticus* 481NCK228 Hlv+ (Helveticin J producer; generously provided by Todd R. Klaenhammer of the Department of Food Science and Microbiology, Southeast Dairy Foods Research Center, NCSU, North Carolina, USA), *Lactobacillus acidophilus* LA5 (lacticin B producer; Christian Hansen, Hørsholm, Denmark) and *Streptococcus salivarius* K12 (salivaricin A and B producer; BLIS Technologies Ltd, Dunedin, NZ).

## Statistical analysis

The statistical analyses were carried out using SPSS version 20 (IBM SPSS, Chicago, IL, USA). Correlations between the bacterial groups (copy numbers per milliliter of colostrum) were evaluated by Spearman’s rank correlation coefficient, and the significance was evaluated by a 2-tailed t test.

DGGE gel images were analyzed in Bionumerics (Applied Maths, Sint-Martens-Latem, Belgium). Band migration was normalized to standard samples present in four lanes of each gel. The standard samples were prepared from ten excised bands distributed evenly throughout the gel gradient. Further normalization was achieved using bands from samples that represented the same migration distance. The Pearson correlation coefficient of DGGE profiles was used as a sample similarity measure, and UPGMA dendrograms were constructed. The bands were classified into band migration categories with a band-matching algorithm within Bionumerics, and the relative peak area of the sample was exported for peaks above 2% of the total fluorescence.

## Results

### DGGE analysis of microbial communities in colostrum samples

**Fig 1:**
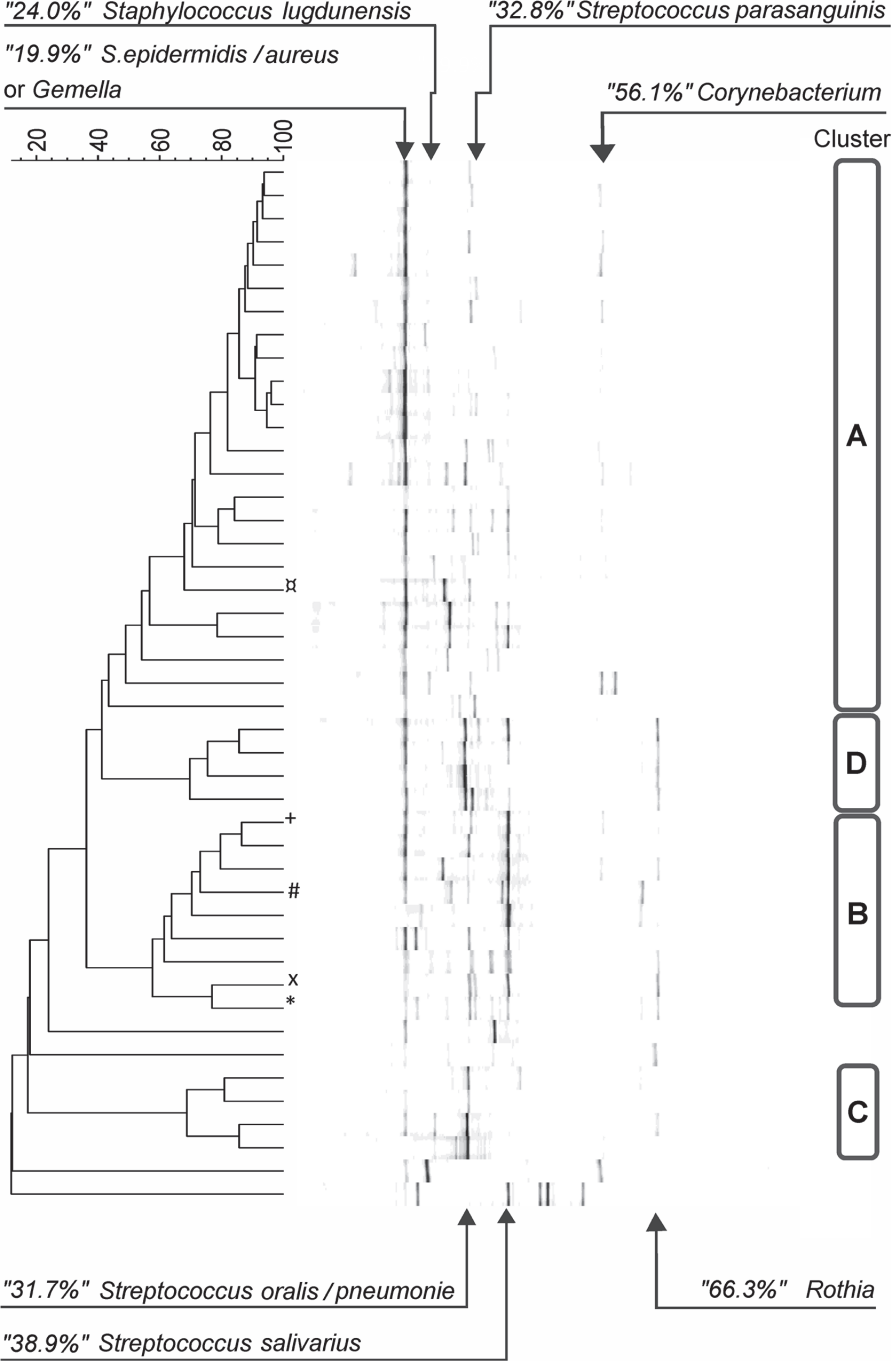
Cluster analysis of DGGE profiles, band migration positions and presumptive identifications. Samples with detected bacteriocin genes are marked on the left as specified in Table 6 (salivaricin A (x, #), salivaricin B (*), streptin (+), cytolysin (¤)).

**Fig 2:**
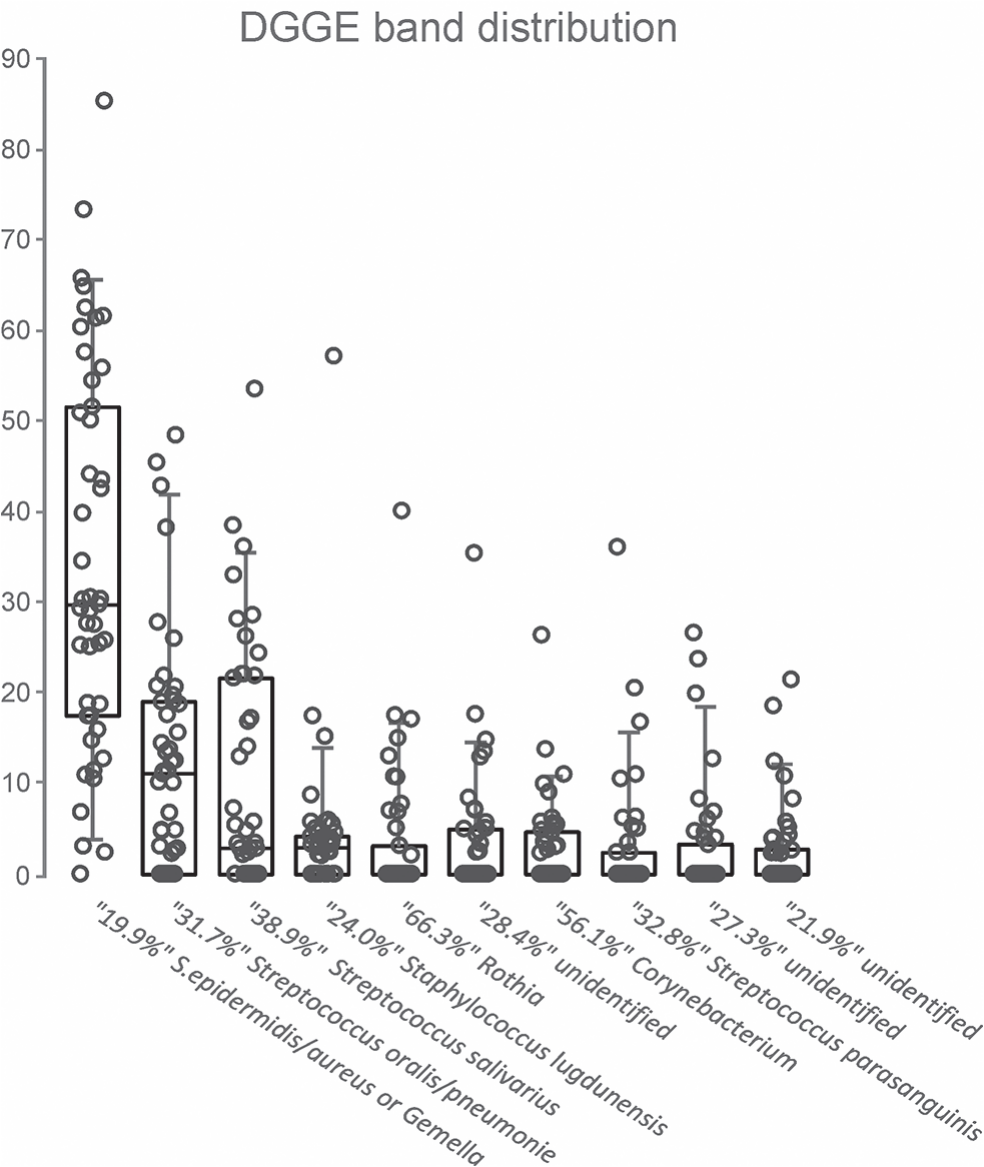
Distribution of the relative fluorescence (abundance, %) of major bands (with signal above 2% of total sample fluorescence) in a sample set together with presumptive band identification. The overlaid box plot represents the values (circles), the box medians and interquartile ranges, and error bars (5^th^ and 95^th^ percentiles).

PCR-DGGE analysis was successfully performed on 45 colostrum samples. In the complete sample set, 40 distinct DGGE band positions were identified with 8.1 bands per sample on average (min 5, max 13, median 8). In most samples, one or two bands dominated, and no more than five bands were prominent in any sample (Fig 1). Bands from the major DGGE migration positions were identified through the excision and sequencing of several samples. The distribution of the relative fluorescence quantity (%) of individual major bands, together with the bands’ presumed identity, is shown in Fig 2. Unambiguous identification was only possible to the genus level, but when possible, the best matching species or two are indicated. While individual bands from almost all the analyzed positions resulted in a clear position identity, two bands excised from the most abundant position (19.9%) produced distinct identities (*Staphylococcus epidermidis/aureus* or *Gemella sp*.). This band was present in 44 samples and represented 34.6% of each sample on average. The next two band positions were identified as *Streptococcus oralis/pneumoniae* and *Streptococcus salivarius* according to their abundance and represented an average of 12.3% and 10.3% of the sample bacterial content, occurring in 33 and 27 subjects, respectively.

Forty-one of the samples formed four distinct clusters using cluster analysis of the DGGE profiles, while four of the samples were not similar to the others. The clusters were associated with the occurrence of three major bands. Samples with *Staphylococcus epidermidis/aureus* or *Gemella sp*. as the predominant band (average abundance 49.0%) formed the largest cluster (cluster A, 24 samples). The second largest cluster (B, 9 samples) was associated with the abundance of *Streptococcus salivarius* (31.0%). The remaining two clusters represented four samples (clusters C and D). While both clusters were characterized by a high abundance of *Streptococcus oralis/pneumoniae*, on average 41.2% (C) and 26.7% (D), they differed in the abundance of *Staphylococcus epidermidis/aureus* or *Gemella sp*. (9.0% and 30.3%). For the latter of these two clusters, the presence of *Rothia sp*. was also characteristic.

### Colostrum qPCR analyses and viable count determination

Real-time PCR quantitation detected bacterial DNA in all the investigated colostrum samples (Table 3). Furthermore, a high prevalence in colostrum samples was confirmed for the *Enterobacteriaceae* group (100%), *Clostridium* cluster XIV (95.6%), *Bacteroides-Prevotella* group (62.2%) and *Bifidobacterium* (53.3%), even though these groups were present in relatively low quantities. *Enterococcus* was detected only in 8.9% (4/45) of the samples, and no lactobacilli were found in the examined colostrum samples.

**Fig 3:**
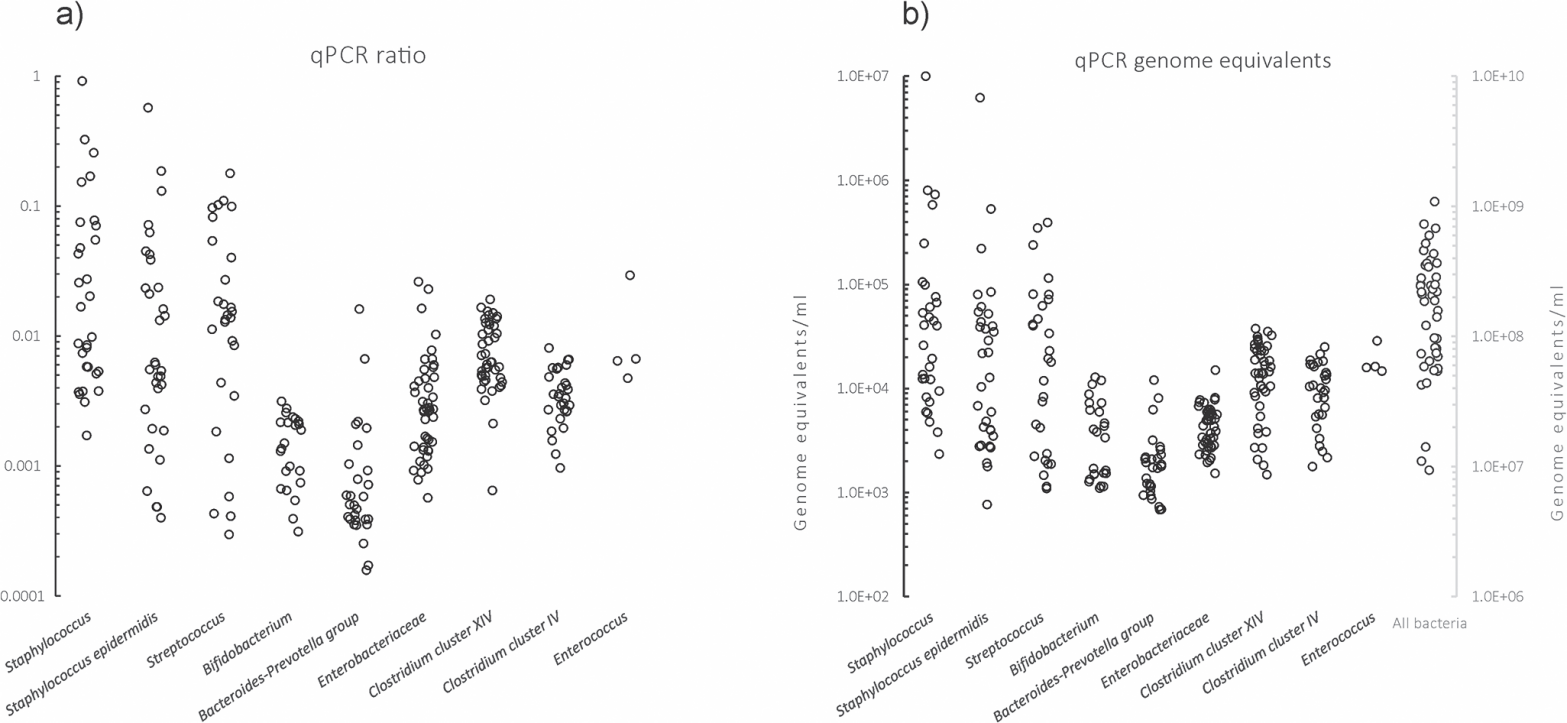
(a) Normalized distribution of detected bacterial groups across the sample set (one dot represents % of the detected group’s specific DNA in relation to all bacterial DNA in the sample). (a, b). (b) Distribution of genome equivalents ml-1 colostrum for detected bacterial groups across the investigated maternal population.

Among the investigated groups, the taxonomic class *Bacilli*, streptococci and staphylococci represented the largest community share in relation to all bacteria. The relative proportions (%) of the quantified group-specific DNA in relation to all the bacterial DNA in the samples with detected bacterial groups are presented in Fig 3(A). Moreover, the bacterial quantity (GE ml^-1^) varied among samples from different mothers; *Staphylococcus epidermidis* and staphylococci varied in the range of 4 logs, streptococci and total bacteria varied in the range of 2 logs, and other researched bacterial groups varied in the range of 1 log (Fig 3(B)).

**Fig 4:**
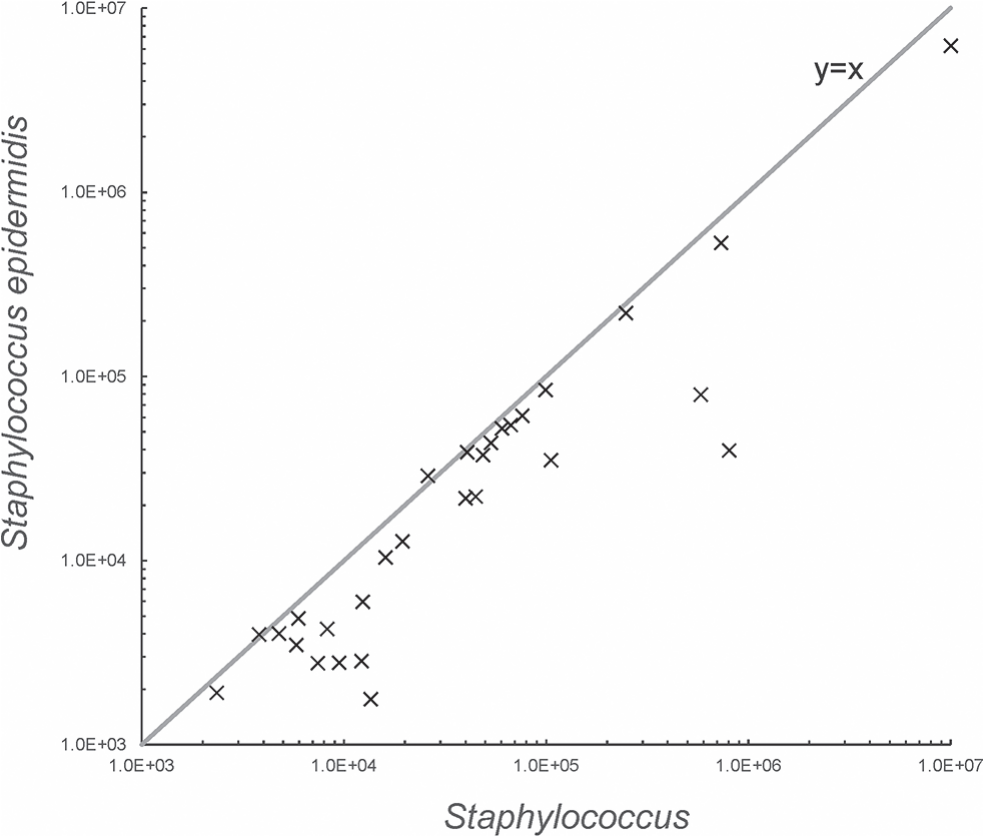
Correlation of *Staphylococcus* and *Staphylococcus epidermidis* in 29 colostrum samples in which both groups were detected. The data are presented as copies (GE) ml^-1^.

The greater part of the quantitatively investigated bacterial groups in colostrum microbiota was correlated with all bacteria (Table 4). The majority of the genus *Staphylococcus* was represented by the species *Staphylococcus epidermidis* (on average 61%, median 65%), and the abundances of genus and species were correlated (Spearman’s rho 0.949; p<0.01) (Fig 4). The presence of staphylococci and *S*. *epidermidis* was confirmed in 29 samples (64%), while in 4 samples, staphylococci were represented only by *S*. *epidermidis* species. In only 2 of the 45 investigated colostrum samples (4.4%), no bacteria were grown on MRS and M17 agar. Most of the colostrum samples contained culturable microbes grown on MRS and M17 agar (predominantly lactic acid bacteria) in the range of 3 log cfu ml^-1^ (Table 5).

**Table 3:** Quantitative PCR analysis of colostrum bacterial DNA in 45 healthy mothers. Data are presented as copies ml-1.

MICROORGANISMS	END CYCLE	PREVALENCE	MEDIAN	SD	RANGE	
					MINIMUM	MAXIMUM
All bacteria	35	100% (45/45)	2.1 x 10^8^	2.2 x 10^8^	9.4 x 10^6^	1.1 x 10^9^
*Staphylococcus*	30	66.7%(30/45)	9.5 x 10^3^	1.5 x 10^6^	1.1 x 10^3^	1.0 x 10^7^
*Staphylococcus epidermidis*	30	71.1%(32/45)	4.0 x 10^3^	9.3 x 10^5^	3.7 x 10^2^	6.2 x 10^6^
*Streptococcus*	30	62.2% (28/45)	2.0 x 10^3^	8.5 x 10^4^	5.1 x 10^2^	4.0 x 10^5^
*Clostridium* cluster XIV	36	95.6% (43/45)	1.4 x 10^4^	1.0 x 10^4^	5.9 x 10^2^	3.8 x 10^4^
*Clostridium* cluster IV	33	64.4% (29/45)	5.3 x 10^3^	6.6 x 10^3^	5.9 x 10^2^	2.5 x 10^4^
*Enterobacteriaceae* group	35	100% (45/45)	4.4 x 10^3^	2.4 x 10^3^	1.5 x 10^3^	1.5 x 10^4^
*Bifidobacterium*	33	53.3% (24/45)	1.2 x 10^3^	3.3 x 10^3^	4.5 x 10^2^	1.3 x 10^4^
*Bacteroides-Prevotella* group	33	62.2%(28/45)	1.0 x 10^3^	2.2 x 10^3^	3.1 x 10^2^	1.2 x 10^4^
*Enterococcus*	36	8.9%(4/45)	3.1 x 10^3^	5.3 x 10^3^	2.4 x 10^3^	2.9 x 10^4^
*Lactobacillus*	30	0% (0/45)	NA	NA	> 4.3 x 10^3^	

**Table 4:** Spearman’s rank correlation coefficients of qPCR targeted bacterial groups (copies ml-1).

		*Staphylococcus*	*S*. *epidermidis*	*Streptococcus*	*Bifidobacterium*	*Bacteroides-Prevotella* group	*Enterobacteriaceae* group	*Clostridium* cluster XIV	*Clostridium* cluster IV	*Enterococcus*	*Lactobacillus*
All bacteria	Correlation Coefficient	0.352(*)	0.331(*)	0.032	0.609(**)	0.669(**)	0.633(**)	0.759(**)	0.809(**)	0.082	NA
	Sig, (2-tailed)	**0.017**	**0.025**	0.833	**0**	**0**	**0**	**0**	**0**	0.59	NA

Correlation is significant (**) at the 0.01 level and (*) at the 0.05 level

**Table 5:** Viable plate count analysis of 45 healthy mothers' colostrums. Data are presented as cfu ml-1.

MEDIUM (lactic acid bacteria)	PREVALENCE (% SAMPLES)			MEDIAN	SD
	COLONIES NOT DETECTED	COUNTABLE PLATES	NON-COUNTABLE PLATES		
	(< 1 x 10^2^)	(1 x 10^2^ <S< 3 x 10^4^)	(>3 x 10^4^)		
MRS AGAR	11.1% (5/45)	77.8%(35/45)	11.1% (5/45)	2.5 x 10^3^	1.9 x 10^4^
M17 AGAR	11.1% (5/45)	66.7% (30/45)	22.2% (10/45)	5.1 x10^3^	2.4 x 10^4^

### PCR screening for bacteriocin genes

Total DNA of the 45 colostrum samples and of the respective MRS and M17 consortia were PCR amplified in search of the following bacteriocin genes: 8 enterococcal (enterocin A, B, P, 31, AS 48, L50A and L50B and cytolysin), 3 lactococcal (nisin, lacticin 481 and lactococcin A), 1 pediococcal (pediocin), 1 staphylococcal (aureocin), 3 streptococcal (salivaricin A and B and streptin) and 9 determinants for bacteriocins usually produced by lactobacilli (acidocin A and B, plantaricin A and S, sakacin P, curvacin A, helveticin J, lactocin 705 and lacticin B). Only cytolysin, streptin and salivaricin A and B were detected in DNA of single samples in colostrum or in MRS consortia (Table 6). In the samples containing specific bacteriocin determinants, PCR quantitation for the respective bacterial group (enterococci and streptococci) showed values higher than the median and the average for the whole set of the investigated colostrum samples (data not shown). Furthermore, the MRS viable count in these samples was also above the median value of the entire set of colostrum samples. Moreover, *Streptococcus salivarius* was identified by DGGE predominantly in 9 samples grouped in cluster B (Fig 1), and in 4 of those samples, streptococcal bacteriocin determinants were also confirmed (Table 6). Interestingly, in those samples, two other bands (*Streptococcus oralis/pneumoniae* and *Staphylococcus epidermidis/aureus* or *Gemella*) also co-occurred.

**Table 6:** PCR detected bacteriocin genes in the DNA of colostrum and respective MRS consortia and sequence similarity values in the NCBI database using BLAST.

Bacteriocin genes (detected)	Fig 1 sample mark	Colostrum	Consortia (MRS)	Query coverage	Identity
Cytolysin	¤	-	+	98%	100%
Salivaricin A	#	+	-	99%	100%
	x	+	+	100%; 100%	99%; 99%
Salivaricin B	*	+/-	+	100%	98%
Streptin	+	+	+	97%; 79%	78%; 82%

^+^ = PCR product of the expected size was amplified and the identity was confirmed by sequence analysis; ^+/-^ = PCR product of the expected size was weakly amplified and the identity was not confirmed by sequence analysis;— = PCR product of the expected size was not detected

## Discussion

In this study, colostrum microbiota was investigated in a population of 45 healthy mothers. Bacterial DNA was successfully recovered from all investigated samples (as affirmed by DGGE and qPCR analysis). The presence of culturable bacteria was confirmed in almost all the samples, supporting the argument that active viable bacteria are a constituent part of colostrum microbiota. Total bacterial load varied by approximately 2 logs among samples of different mothers. Quantitative and qualitative differences in the microbial population of colostrum samples are most likely the outcome of the various backgrounds of the mothers (environmental, genetic, physiological, dietary, etc.) [2, 28], which are also tightly associated with colostrum composition and its bioactive potential. Imbalances in breast microbial communities were reported to lead to mastitis [29] or may even be associated with breast cancer [30]. In our study, the abundance correlations of targeted bacterial groups associated with the mammary gland exhibited some similarities in the microbial consortia of healthy mothers, which could play a role in the maintenance of homeostasis.

DGGE analysis disclosed bands identified as *Staphylococcus epidermidis/aureus* and *Gemella*, as the common feature for the majority of colostrum samples. In approximately one third of the samples, this band was accompanied by either of the two streptococci bands recognized as *Streptococcus oralis/pneumoniae* and *Streptococcus salivarius*, respectively. A recent study employing *in vitro* assays with *Streptococcus oralis* and *Streptococcus salivarius* reported that these two bacterial species form biofilms on epithelial cells and offer protection from respiratory infection by airway pathogenic streptococci in a strain specific manner, possibly through bacteriocin production [31].

*S*. *salivarius*, recognized as a pioneer colonizer of oral surfaces, was reported [32] to be isolated from human colostrum. It was also identified as a potential oral probiotic because the strain exhibited many probiotic properties, such as bacteriocin production, biofilm formation and exopolysaccharide synthesis. In our set of colostrums, 4 out of 9 samples with abundant *Streptococcus salivarius* also contained bacteriocin determinants known to be involved in inter- and intra-species inhibition. Bacteriocin genes were confirmed in the DNA of culturable microbiota in 3 samples. Enterococci, however, were found only in 8.9% (4/45) of the colostrum samples, and as opposed to the study of Jímenez et al. [15], one of our samples’ viable consortia also contained a cytolysin determinant. Finally, in the samples identified to contain specific bacteriocin gene, PCR quantitation of the respective bacterial group (streptococci and enterococci, respectively) quantity was above the median and the average value for the whole set of the investigated colostrums. This suggests that the capability of the respective species to produce bacteriocins could have contributed to the quantitative enrollment of streptococci and enterococci in these communities.

Our findings by DGGE are in agreement with the current reports summarized by Quigley et al. [33] acknowledging *Staphylococcus*, *Streptococcus* and *Corynebacterium* species as the most prevalent bacterial populations consistently found across all human milk samples. Additionally, *Rothia* and *Gemella* members identified in our sample set were reported to be found in human milk by culture independent techniques.

The majority of the genus *Staphylococcus* in our study was represented by the species *Staphylococcus epidermidis* (on average 61%), and the abundance of the genus and species was significantly correlated. In four samples, the *Staphylococcus* group was composed entirely of *Staphylococcus epidermidis*, a commensal species that normally inhabits the skin and mucosal surfaces. Jiménez et al. [15] proposed that a high predominance of *Staphylococcus epidermidis* in human milk could result from its adhesion traits contributing to the staphylococcal attachment to areola and ducts in the mammary gland. Furthermore, *S*. *epidermidis* strains characterized in their study were low in virulence determinants and low in antibiotic resistance. This species was also more frequently found in the fecal microbiota of breast-fed infants compared to formula-fed babies, indicating its presence in the initial colonization of the neonate's gut [34]. In our study, *Staphylococcus epidermidis* and staphylococci GE ml^-1^ varied greatly (in the range of 4 logs) among samples from different mothers. Research conducted on the breast milk of women with lactational infectious mastitis [18] found DGGE profiles to be host-specific. *S*. *epidermidis* was the most widely distributed species, even outnumbering *S*. *aureus*. While representatives of the genera *Gemella*, *Corynebacterium*, *Rothia*, and *Streptococcus* were also found, staphylococci are one of the main etiological agents causing human lactational mastitis. The outgrowth of staphylococci may alter the composition of milk microbiota and consequently provoke a dysbiotic process in a predisposed host. A recent study [35] proposed that a woman’s specific milk oligosaccharide profile might be involved in lactational mastitis by stimulating the growth of staphylococci.

Streptococci are one of the other predominant representatives of the breast milk microbiota [10–12] and are also common mastitis-causing agents [29]. Interestingly, our study showed that both bacterial groups in healthy mothers were quantitatively correlated (p<0.01) (data not shown). It should be emphasized, however, that only specific strains with particular virulent characteristics within the discussed species and genera domain could lead to certain clinical outcomes in the exposed host. The quantitative results confirmed that the ratio (%) of streptococci and staphylococci relative to the total bacterial genome equivalents was the highest of all the investigated bacterial targets. Additionally, DGGE analysis (with different universal primer sets) and band sequencing asserted these two groups as major colostrum representatives. However, the relative abundance of these two groups was profoundly underestimated. Similar observations have been reported previously [16, 36]. A bias in the total bacterial load estimation, due to chromosomal standard DNA and to errors made in approximating the higher taxa genome equivalents, could have contributed to the results obtained. Finally, the sample type and the low amount of sample bacterial DNA increased the possibility of bias detection with the universal primer sets used because of co-extracted eukaryotic host DNA. Recent reports have indicated that only carefully selected primer pairs among the commonly used primers can approximate metagenomic approaches and avoid accumulative bias in the relative abundances of the community structure [37–39]. In our study, this biased effect was considered equal for all absolute values of the different samples being compared in the analysis.

High prevalences of *Enterobacteriaceae* (100%), *Clostridia* (95.6%), *Bacteroides-Prevotella* (62.2%) and *Bifidobacterium* (53.3%) were confirmed in colostrum samples, even though these groups were represented at relatively low quantities. Detection of these bacteria is in accordance with recent research [7] suggesting that gut-associated obligate anaerobic genera are shared among ecological niches in the initial stage of a neonate's gut colonization. Furthermore, *Enterobacteriaceae* detected in colostrum samples are in accordance with the expected pattern of initial colonization of the infant gut by facultative anaerobes that create an oxygen-reduced environment advantageous for further succession by the anaerobic groups *Bacteroides*, *Bifidobacterium* and *Clostridium*, which occurs by the end of the first week. Members of these groups decrease later, and bifidobacteria usually become dominant [40, 41]. Minute quantities of bacterial DNA resulting from environmental contamination during sampling could also have contributed on a small scale to the results obtained. Finally, it should be noted here that the composition of detected microbiota largely depends on the study techniques used. Biases leading to underestimation of the relative abundances of the Gram-negative bacteria were reported to be introduced throughout the procedure of the molecular approaches [42]. Taking into account the rather limited species diversity and microbial quantities expected in our sample type according to the literature, the traditional technologies (DGGE, qPCR and culturomics) were considered as the best complementary alternatives to overcome the detection threshold inherent in metagenomic approaches [43] and to capture viable and functional part of the lactic acid microbiota and its bacteriocinogenic potential. In contrast to Ozgun and Vural [14], who identified by culture-based methods 5 different species of lactobacilli in 100 colostrum samples, in our study, no lactobacilli or their distinctive bacteriocin genes were found.

## Conclusions

This work offers insight into the colostrum microbial community composition of healthy Slovenian lactating mothers and describes the bacterial distribution and abundance, quantitative bacterial associations and bacteriocin gene prevalence. To our knowledge, this is the first study using a combined qPCR and DGGE approach in studying human colostrum microbiota. Our study confirmed the presence of staphylococci and streptococci as a common feature of most investigated colostrums in healthy mothers. The prevalence of targeted bacteriocin genes was limited to streptococci representatives in single individuals only, indicating that the occurrence of the investigated bacteriocin genes in colostrum was not a common phenomenon. The detected bacteriocin genes indicate the production of bacteriocins that may offer select species a competitive advantage in colonizing an infant's oral mucosa, given that many oral bacteria use bacteriocin-like compounds to inhibit other species. Abundance correlations of the targeted bacterial groups associated with the mammary gland revealed common themes in some microbial consortia, which could play a role in the maintenance of homeostasis. This study provides evidence for the potential of colostrum to supply a range of microbes that support mammary gland health in mothers and to deliver low amounts of gut-associated genera to their newborns.

## Supporting Information

S1 Table Primer sequences of 25 targeted bacteriocin genes and their references. (DOC)
