# Correction: Colostrum of Healthy Slovenian Mothers: Microbiota Composition and Bacteriocin Gene Prevalence

**DOI:** 10.1371/journal.pone.0132201

**Published:** 2015-06-29

**Authors:** Tanja Obermajer, Luka Lipoglavšek, Gorazd Tompa, Primož Treven, Petra Mohar Lorbeg, Bojana Bogovič Matijašić, Irena Rogelj

The image for Fig 3 is incorrect. Please see the corrected Fig 3 here.

**Fig 3:**
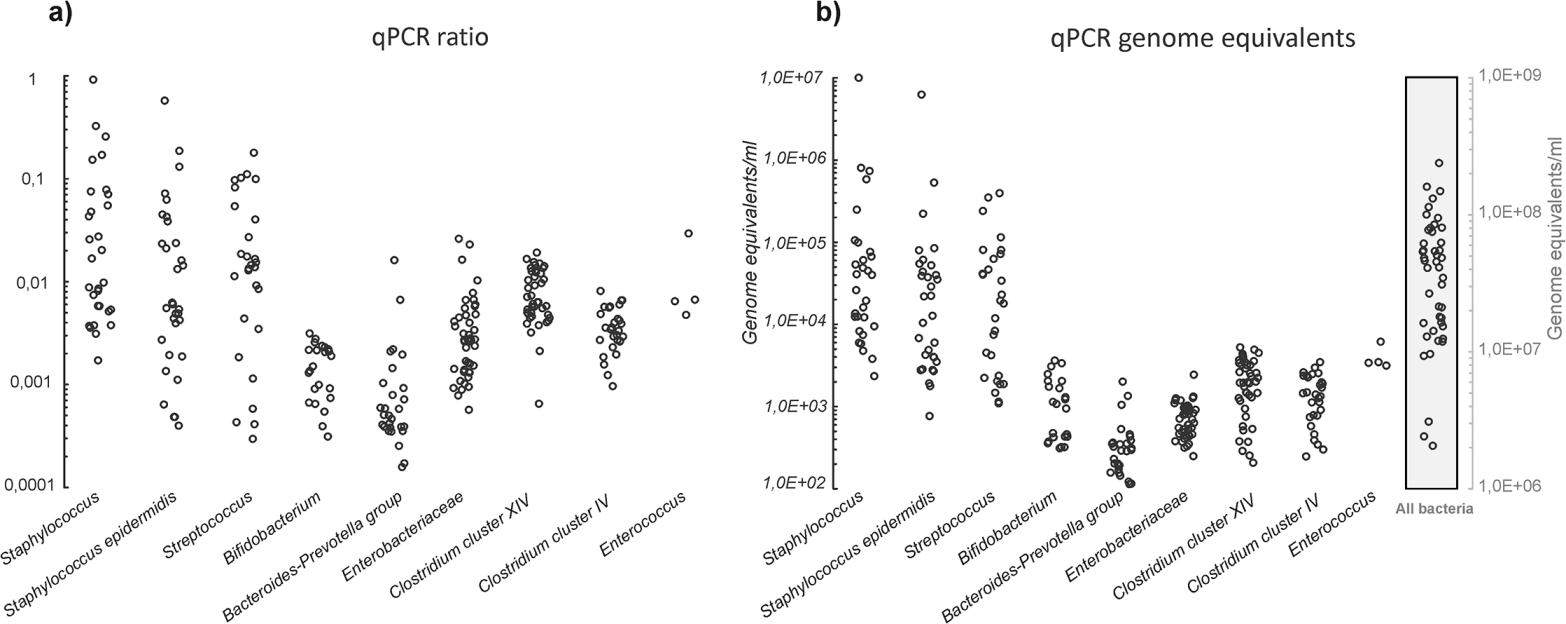
(a) Normalized distribution of detected bacterial groups across the sample set (one dot represents % of the detected group’s specific DNA in relation to all bacterial DNA in the sample). (a, b). (b) Distribution of genome equivalents ml-1 colostrum for detected bacterial groups across the investigated maternal population.
