# Supplementary material for: Colostrum of Healthy Slovenian Mothers: Microbiota Composition and Bacteriocin Gene Prevalence
Source: PLoS One. 2015 Apr 28;10(4):e0123324. doi: 10.1371/journal.pone.0123324 (PMC4412836; doi:10.1371/journal.pone.0123324)
Supplement: S1 Table — (DOC) [file pone.0123324.s001.doc]

**Colostrum of Healthy Slovenian Mothers: Microbiota Composition and Bacteriocin Gene Prevalence**

Tanja Obermajer^1*^, Luka Lipoglavšek^2^, Gorazd Tompa^1^, Primož Treven^1^, Petra Mohar Lorbeg^1^, Bojana Bogovič Matijašić^1^, Irena Rogelj^1^

^1^ Institute of Dairy Science and Probiotics, Department of Animal Science, Biotechnical faculty, University of Ljubljana, Domžale, Slovenia

^2^ Department of Animal Science, Biotechnical faculty, University of Ljubljana, Domžale, Slovenia

* Corresponding author

E-mail: [tanja.obermajer@bf.uni-lj.si](mailto:tanja.obermajer@bf.uni-lj.si) (TO)

**S1 Table.** Primer sequences of 25 targeted bacteriocin genes and their references.

| Primer sequence (5’ to 3’) | Target bacteriocin | Reference^a^ |
| --- | --- | --- |
| f: 5’-ggt acc act cat agt gga aa-3’; r: 5’-ccc tgg aat tgc tcc acc taa-3’ | enterocin A | [52] |
| f: 5’-caa aat gta aaa gaa tta agt acg-3’; r: 5’-aga gta tac att tgc taa ccc-3’ | enterocin B | [52] |
| f: 5’-gct acg cgt tca tat ggt aat-3’; r: 5’-tcc tgc aat att ctc ttt agc-3’ | enterocin P | [52] |
| f: 5’-cct acg tat tac gga aat ggt-3’; r: 5’-gcc atg ttg tac cca acc att-3’ | enterocin 31 | [52] |
| f: 5’-gag gag tat cat ggt taa aga-3’; r: 5’-ata ttg tta aat tac caa-3’ | enterocin AS 48 | [52] |
| f: 5’-atg gga gca atc gca aaa tta-3’; r: 5’-ttt gtt aat tgc cca tcc ttc-3’ | enterocin L50A | [52] |
| f: 5’-atg gga gca atc gca aaa tta-3’; r: 5’-tag cca ttt ttc aat ttg atc-3’ | enterocin L50B | [52] |
| f: 5’- ggc ggt att ttt act gga gtn-3’; r: 5’- cct act cct aag cct atg gta-3’ | cytolysin | [52] |
| f: 5’-aag aat ctc tca tga gt-3’; r: 5’-cca tgt ctg aac taa ca-3’ | nisin | [53] |
| f: 5’-tct gca ctc act tca tta gtt a-3’; r: 5’-aag gta att aca cct ctt tta-t3’ | lacticin 481 | [53] |
| f: 5’-caa tca gta gag tta tta aca ttt g-3’; r: 5’-gat tta aaa aga cat tcg ata att at-3’ | lactococcin A | [53] |
| f: 5’-tgg tgt gca ttg tac t-3’; r: 5’-ttg atc ggc aac gat t-3’ | acidocin A | [54] |
| f: 5’-aga tgc agt ggc ttc t-3’; r: 5’-cca tgc agg taa tgt c-3’ | acidocin B | [54] |
| f: 5’-atg aaa att caa att aaa ggt atg aag c-3’; r: 5’-tta cca tcc cca ttt ttt aaa cag ttt c-3’ | plantaricin A | [27] |
| f: 5’-aay aar yti gci tay aay atg-3’; r: 5’-gcy tty aar rai ccy tci cc-3’ | plantaricin S | [55] |
| f: 5’-atg gaa aag ttt att gaa tta-3’; r: 5’-tta ttt att cca gcc agc gtt-3’ | sakacin P | [55] |
| f: 5’- gta aaa gaa tta agt atg aca-3’; r: 5’-tta cat tcc agc taa acc act-3’ | curvacin A | [55] |
| f: 5’-aga cat ggg aat ttg ctg gt-3’; r: 5’- ggc gcg att caa gta gga ta –3’ | helveticin J | [20] |
| f: 5’- tcc aag acg tcc ctt ttt gt -3’; r: 5’- tta cag gtg gtg gat ttt gg-3’ | lactocin 705 | [20] |
| f: 5'- ttg agt tat gta att ggt gga g-3'; r: 5'- ctg ctg agc ctt tga taa cg-3' | lactacin B | [56] |
| f: 5'- gta gaa aat att tac tac ata ct-3'; r: 5'- gtt aaa gta ttc gta aaa ctg atg-3' | salivaricin A | [57] |
| f: 5'- gtg aat tct ctt caa gaa ttg act ctt-3'; r: 5'- aaa ata ttc ata ccg ctc ttc c-3' | salivaricin B | [57] |
| f: 5'- ctt ttt ttc tga aca rat tct gaa ct-3'; r: 5'- taa agg agc aaa cta aaa wca gtc tac t-3' | streptin | [57] |
| f: 5'- gat taa acc tta taa tag a-3'; r: 5'- cta ata ata aaa tat taa caa-3' | aureocin | [58] |
| f: 5'- aaa gaa ttc atg aaa aaa att gaa aaa tta act g-3'; r: 5'- aaa ggt acc cta gca ttt atg att acc ttg atg tcc-3' | pediocin | [59] |

^a^[20] Trmčić A, Obermajer T, Rogelj I, Bogovič Matijašić B (2008) Short Communication: Culture-Independent Detection of Lactic Acid Bacteria Bacteriocin Genes in Two Traditional Slovenian Raw Milk Cheeses and Their Microbial Consortia. J Dairy Sci 91: 4535-4541. doi: 10.3168/jds.2008-1396

^a^[27] Maldonado A, Jimenez-Diaz R, Ruiz-Barba JL (2004) Induction of Plantaricin Production in Lactobacillus plantarum NC8 after Coculture with Specific Gram-Positive Bacteria Is Mediated by an Autoinduction Mechanism. J Bacteriol 186: 1556-1564. doi: 10.1128/jb.186.5.1556-1564.2004

^a^[52] De Vuyst L, Foulquié Moreno MR, Revets H (2003) Screening for enterocins and detection of hemolysin and vancomycin resistance in enterococci of different origins. Int J Food Microbiol 84: 299-318. doi: 10.1016/s0168-1605(02)00425-7

^a^[53] Rodriguez E, Gonzalez B, Gaya P, Nunez M, Medina M (2000) Diversity of bacteriocins produced by lactic acid bacteria isolated from raw milk. Int Dairy J 10: 7-15. doi: 10.1016/S0958-6946(00)00017-0

^a^[54] Čanžek Majhenič A, Bogovič Matijašić B, Rogelj I (2003) Chromosomal location of the genetic determinants for bacteriocins produced by Lactobacillus gasseri K7. J Dairy Res 70: 199-203. doi: 10.1017/s0022029903006162

^a^[55] Remiger A, Ehrmann MA, Vogel RF (1996) Identification of bacteriocin-encoding genes in lactobacilli by polymerase chain reaction (PCR). Syst Appl Microbiol 19: 28-34.

^a^[56] Tabasco R, García-Cayuela T, Peláez C, Requena T (2009) Lactobacillus acidophilus La-5 increases lactacin B production when it senses live target bacteria. Int J Food Microbiol 132: 109-116. doi: 10.1016/j.ijfoodmicro.2009.04.004

^a^[57] Wescombe PA, Burton JP, Cadieux PA, Klesse NA, Hyink O, Heng NCK, et al. (2006) Megaplasmids encode differing combinations of lantibiotics in Streptococcus salivarius. Antonie Van Leeuwenhoek 90: 269-280. doi: 10.1007/s10482-006-9081-y

^a^[58] Brito MAVP, Somkuti GA, Renye Jr JA (2011) Isolation of Bacteriocin-Producing Staphylococci from Brazilian Cheese. J Food Saf 31: 365-370. doi: 10.1111/j.1745-4565.2011.00308.x

^a^[59] Millette M, Dupont C, Shareck F, Ruiz MT, Archambault D, Lacroix M (2008) Purification and identification of the pediocin produced by Pediococcus acidilactici MM33, a new human intestinal strain. J Appl Microbiol 104: 269-275. doi: 10.1111/j.1365-2672.2007.03583.x
